# Enhancing wound healing in otorhinolaryngology with autologous platelet concentrates: a systematic review of clinical outcomes

**DOI:** 10.1017/S0022215126104320

**Published:** 2026-04

**Authors:** Rizki Ekaputra Handoko, Loeki Enggar Fitri, Mirta Hediyati Reksodiputro, Dhelya Widasmara

**Affiliations:** 1 Doctoral Program in Medical Science, Faculty of Medicine, Universitas Brawijaya, Malang, Indonesia; 2 Department of Otorhinolaryngology-Head and Neck Surgery, Faculty of Medicine, Universitas Brawijaya/Dr. Saiful Anwar General Hospital, Malang, Indonesia; 3 Department of Clinical Parasitology, Faculty of Medicine, Universitas Brawijaya, Malang, Indonesia; 4 Department of Otorhinolaryngology-Head and Neck Surgery, Faculty of Medicine, Universitas Indonesia, Indonesia; 5 Department of Dermatology Venereology and Aesthetics, Faculty of Medicine, Universitas Brawijaya/Dr. Saiful Anwar General Hospital, Malang, Indonesia

**Keywords:** immunomodulation wound healing, platelet-rich fibrin, platelet-rich plasma, otorhinolaryngologic surgical procedures, post-operative complications

## Abstract

**Objectives:**

This study aimed to evaluate the clinical efficacy and potential immunomodulatory effects of autologous platelet concentrates in promoting tissue regeneration and enhancing post-operative recovery in otorhinolaryngology.

**Methods:**

A systematic search of PubMed, Scopus and the Cochrane Library was conducted through November 2025, focusing on randomised controlled trials and prospective studies using autologous platelet concentrates in otological, rhinological and laryngological surgery.

**Results:**

Twelve studies met the inclusion criteria. Autologous platelet concentrates improved tissue healing, most notably by increasing graft uptake rates in myringoplasty. Recent trials (2024–2025) also reported reduced post-operative pain and mucosal oedema following tonsillectomy and tympanoplasty.

**Conclusion:**

Autologous platelet concentrates appear to be effective adjuncts for enhancing regeneration and reducing post-operative morbidity in ear, nose, and throat procedures. These benefits likely involve downregulation of pro-inflammatory cytokines (e.g., interleukin-6) and modulation of matrix metalloproteinases (e.g., matrix metalloproteinase-9). Standardised biomarker-based studies are needed to confirm these mechanisms.

## Introduction

Wound healing within the field of otorhinolaryngology presents significant clinical challenges due to the complex anatomical structures and the critical functional roles of the tissues involved. Successful tissue repair in the mucosal surfaces of the ear, nose and throat is essential for restoring physiological functions such as hearing, breathing and phonation. While traditional surgical techniques often achieve acceptable repair rates, the healing process can be prolonged and is frequently complicated by factors such as infection, graft failure, excessive fibrosis or persistent post-operative pain.^1^^,^^2^ These complications not only delay recovery but also significantly impact the patient’s quality of life.

Autologous platelet concentrates (APCs), including platelet-rich plasma (PRP) and platelet-rich fibrin (PRF), have emerged as promising adjunctive therapies aimed at enhancing wound healing outcomes. These preparations use the patient’s own blood to deliver a supraphysiological concentration of platelets, which, upon activation, release a substantial pool of bioactive growth factors and cytokines.^3^ Initially, the clinical rationale for using APCs focused primarily on their structural benefits specifically their ability to accelerate graft uptake and promote epithelialisation through the release of growth factors such as platelet-derived growth factor (PDGF) and transforming growth factor-beta 1 (TGF-β1).^3^^,^^4^

However, recent clinical observations suggest that the therapeutic utility of APCs extends beyond mere structural repair. Increasing evidence indicates that APCs possess significant immunomodulatory properties that may directly influence post-operative morbidity. Recent randomised controlled trials have highlighted a reduction in post-operative pain and mucosal oedema as critical outcome measures, suggesting a complex biological interplay.^4^^,^^5^ Theoretically, this modulation is driven by the regulation of the inflammatory microenvironment, where APCs may help balance pro-inflammatory cytokines, such as interleukin-6 (IL-6), with tissue-remodeling enzymes like matrix metalloproteinases (MMP-9).^6^ Despite the widespread clinical adoption of APCs in otorhinolaryngological surgeries, the correlation between these specific clinical improvements and their underlying biological mechanisms remains to be fully elucidated.

The current literature reveals heterogeneity in clinical outcomes, likely attributable to variations in preparation protocols and surgical applications.^7^ Furthermore, the emergence of new randomised controlled trials (RCTs) in 2024 and 2025 necessitates an updated synthesis of the evidence to validate these evolving clinical perspectives.^8^^,^^9^ Therefore, this systematic review aims to comprehensively evaluate the clinical efficacy of autologous platelet concentrates on tissue regeneration outcomes in otorhinolaryngology. Additionally, it seeks to discuss the potential biological mechanisms, including immunomodulation and growth factor activity, that underlie these observed therapeutic benefits.

## Methods

### Literature search strategy

This systematic review was conducted in accordance with the Preferred Reporting Items for Systematic Reviews and Meta-Analyses (PRISMA) guidelines. The review protocol focused on evaluating the clinical efficacy of APCs in enhancing tissue regeneration and reducing post-operative morbidity in otorhinolaryngological procedures.

Search Strategy A comprehensive systematic literature search was performed across three major electronic databases: PubMed, ScienceDirect and the Cochrane Library. To ensure the inclusion of the most recent evidence, the search spanned from database inception up to November 2025. The search strategy used a combination of Medical Subject Headings (MeSH) terms and free-text keywords, including: *“Platelet-rich plasma”* OR *“PRP”, “Platelet-rich fibrin”* OR *“PRF”, “Autologous platelet concentrates”, “Otorhinolaryngology”, “ENT surgery”, “Tympanoplasty”, “Myringoplasty”, “Endoscopic sinus surgery”, “Tonsillectomy”, “Wound healing”, “Tissue regeneration”* and *“Graft uptake”*. Reference lists of retrieved articles and relevant systematic reviews were also manually screened to identify additional eligible studies.

### Eligibility criteria

Studies were deemed eligible for inclusion if they met the following criteria: (1) study design: RCTs and prospective comparative studies; (2) population: patients undergoing otorhinolaryngological surgical procedures, including otological, rhinological and laryngological interventions; (3) intervention: application of autologous platelet concentrates (e.g., PRP, PRF) compared with conventional treatment or placebo; and (4) outcomes: assessment of clinical wound healing parameters (e.g., graft uptake rates, epithelialisation) or post-operative morbidity outcomes, specifically pain scores and mucosal oedema. Studies were excluded if they were case reports, case series with fewer than 10 patients, reviews or editorials. Animal and in vitro studies were also excluded from the primary clinical analysis, although relevant preclinical data were consulted to support the discussion of biological mechanisms. Furthermore, studies using non-autologous or synthetic platelet products were omitted from this review.

### PICO framework

The present systematic review was structured based on the PICO (Population, Intervention, Comparison, Outcome) framework to ensure a focused and systematic analysis of the available evidence. The components of the PICO framework are summarised in Table 1.

**Table 1.** PICO framework

Component	Description
Population (P)	Patients of any age or gender undergoing surgical procedures in the field of otorhinolaryngology (otology, rhinology or laryngology).
Intervention (I)	Topical or injectable application of autologous platelet concentrates (APCs), including platelet-rich plasma (PRP), platelet-rich fibrin (PRF) or concentrated growth factors (CGF).
Comparison (C)	Control groups receiving conventional wound care, placebo, saline irrigation or no additional intervention.
Outcome (O)	*Primary outcomes:* Structural tissue regeneration, defined as graft uptake rates, complete perforation closure or mucosal epithelialisation time.
	*Secondary outcomes:* Post-operative morbidity and recovery parameters, specifically including Visual Analog Scale (VAS) scores for pain, reduction in mucosal oedema and incidence of post-operative bleeding or infection.

### Study selection

**Figure 1.**
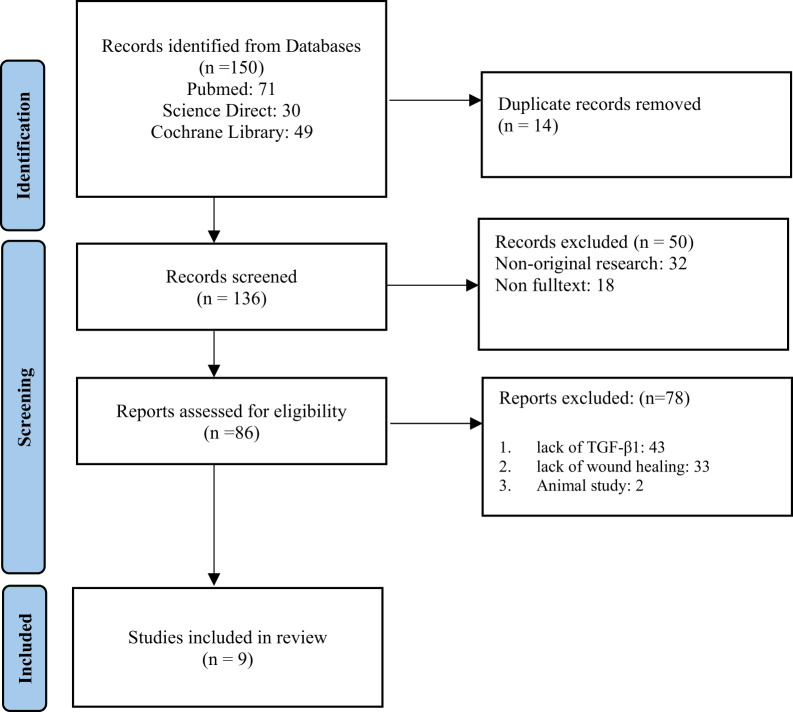
PRISMA (Preferred Reporting Items for Systematic Reviews and Meta-Analyses) flow diagram of the study selection process.

All identified records from the electronic databases were imported into a reference management software to facilitate the screening process. After the removal of duplicates, two independent reviewers screened the titles and abstracts of the retrieved records against the pre-defined eligibility criteria. Articles considered potentially relevant were retrieved in full text for a detailed assessment. The final decision on inclusion was made based on the full-text review. Any discrepancies or disagreements between the reviewers regarding study selection were resolved through discussion and consensus. This comprehensive selection process is illustrated in the PRISMA flow diagram (Figure 1).

### Data extraction

Data extraction was performed independently by two reviewers using a pre-designed standardised form to ensure consistency and accuracy. The following variables were extracted from each included study: (1) study characteristics: first author, year of publication, country, study design and sample size; (2) intervention details: type of autologous platelet concentrate used (e.g., PRP, PRF), preparation protocols (centrifugation speed and time) and method of application; (3) clinical outcomes: primary data on structural tissue regeneration (e.g., graft uptake rates, perforation closure and epithelialisation time) and secondary data on post-operative morbidity (e.g., Visual Analog Scale [VAS] pain scores, mucosal oedema); and (4) proposed mechanisms: any reported or hypothesised biological pathways, including growth factor activity and immunomodulation. Any discrepancies in the extracted data were resolved through discussion between the reviewers until a consensus was reached.

### Risk of bias and study quality assessment

The methodological quality of the included studies was assessed independently by two reviewers using tools appropriate for each study design. For RCTs, the Cochrane Risk of Bias Tool (RoB 2) was employed to evaluate domains such as randomisation process, deviations from intended interventions, missing outcome data, measurement of the outcome and selection of the reported result. For non-randomised prospective studies, the Newcastle–Ottawa Scale (NOS) was used to assess the selection of study groups, comparability of the groups and ascertainment of the outcome of interest. Any disagreements in the quality assessment were resolved through discussion and consensus.

### Data synthesis

Given the significant heterogeneity across the included studies regarding study designs, types of platelet concentrates (PRP, PRF and CGF), preparation protocols and surgical applications, a statistical meta-analysis was not feasible. Consequently, a narrative synthesis was conducted to summarise the findings. The data were tabulated and categorised according to the anatomical sub-specialty (otology, rhinology and laryngology/head and neck). Clinical outcomes were synthesised descriptively, focusing on the direction of effect (positive, negative or neutral) regarding tissue regeneration and post-operative morbidity. Additionally, reported biological mechanisms were qualitatively analysed to identify common molecular pathways across the studies.

## Results

### Article search results

The systematic literature search conducted across three major electronic databases: PubMed (*n* = 142), Scopus (*n* = 161) and the Cochrane Library (*n* = 12) yielded a total of 315 records. After removing 105 duplicates, 210 records remained for screening based on titles and abstracts. During this initial screening phase, 170 records were excluded, primarily because they focused on oral and maxillofacial/dental procedures (e.g., alveolar ridge preservation, dental extraction) rather than specific otorhinolaryngological applications, or were animal and in vitro studies. Consequently, 40 full-text articles were retrieved and assessed for eligibility. Upon detailed examination, 28 articles were excluded due to reasons such as: insufficient quantitative data on specific clinical wound healing or morbidity outcomes (*n* = 14), retrospective or non-compliant study designs (*n* = 8) and the use of synthetic or non-autologous platelet products (*n* = 6). Ultimately, 12 studies met the inclusion criteria and were included in the qualitative synthesis.

### Risk of bias assessment

The methodological quality of the nine included RCTs was evaluated using the Cochrane Risk of Bias 2 (RoB 2) tool. The assessment revealed a variable risk profile across the studies. The domains related to “randomisation process” and “deviations from intended interventions” were the most frequent sources of bias, largely due to the inherent difficulty in blinding the surgical personnel to the intervention. However, recent trials, specifically Steiner *et al.* (2025) and Wiriyaamornchai *et al.* (2025) demonstrated a “low risk” of bias, employing rigorous double-blind protocols where both patients and outcome assessors were masked to the treatment allocation.^4^^,^^5^ A summary of the risk of bias distribution for RCTs is presented in Table 2.

**Table 2.** Risk of bias assessment of included randomised controlled trials using Cochrane RoB 2 Tool

Study ID	D1	D2	D3	D4	D5	Overall Risk
Vignesh et al. (2022)^2^	Some	Some	Low	Some	Low	Some Concerns
Sari et al. (2021)^3^	Low	Low	Low	Low	Low	Low
Steiner et al. (2025)^4^	Low	Low	Low	Low	Low	Low
Wiriyaamornchai et al. (2025)^5^	Low	Low	Low	Low	Low	Low
Reksodiputro et al. (2021)^11^	Some	Low	Low	Low	Low	Some Concerns
Oflaz et al. (2024)^12^	Low	Some	Low	Low	Low	Some Concerns
Katz et al. (2025)^13^	Some	Some	Low	Some	Low	Some Concerns
Mandour et al. (2023)^15^	Low	High	Low	Some	Low	High
Salaheldin (2012)^16^	High	High	Low	High	Some	High

For the three non-randomised prospective studies: Aboelnaga *et al.*, 2022; Saini *et al.*, 2024; Mackay and Allen, 2024, quality was assessed using the NOS.^1^^,^^9^^,^^10^ These studies generally achieved good quality scores (seven to eight out of nine stars), particularly in the Selection and Outcome domains, indicating representative patient cohorts and accurate follow-up. However, they scored lower in the Comparability domain due to the lack of stringent matching for confounding variables. The quality assessment for these studies is detailed in Table 3.

**Table 3.** Quality assessment of non-randomised prospective studies using Newcastle–Ottawa Scale

Study ID	Selection (Max 4*)	Comparability (Max 2*)	outcome (Max 3*)	total score	Quality level
Aboelnaga *et al.* (2022)^1^	***	*	***	7/9	Good
Saini *et al.* (2024)^9^	****	*	***	8/9	Good
Mackay & Allen (2024)^10^	***	*	***	7/9	Good

### Study characteristics

The 12 included studies varied in design, with most being prospective and randomised (e.g. Steiner *et al.*, 2025; Wiriyaamornchai *et al.*, 2025; Reksodiputro *et al.*, 2021; Oflaz çapar *et al.*, 2024).^4^^,^^5^^,^^11^^,^^12^ While some were non-randomised prospective studies (e.g., Mackay and Allen, 2024).^10^ Sample sizes were heterogeneous, ranging from 20 to 320 participants, with smaller studies often serving as pilot trials and larger cohorts providing more robust clinical data.^13^^,^^14^ The characteristics of the included studies are summarised in Table 4.

**Table 4.** Summary of study characteristics and clinical outcomes

Reference	Study design	Sample size	Procedure	APC type	Clinical wound healing outcomes	Proposed biological mechanism
Aboelnaga et al. (2022)^1^	Prospective Comparative	50	Myringoplasty	PRF	Higher success rate of closure (92% vs 80%), though statistically non-significant.	Attributed to rich content of growth factors and their sustained release supporting epithelialization.
Vignesh et al. (2022)^2^	RCT	76	Myringoplasty	PRP	Improved graft uptake (97.4% vs 89.5%) and reduced graft migration.	Autologous serum rich in proteins promotes healing through fibroblast recruitment and collagen synthesis.
Sari et al. (2021)^3^	Prospective RCT	50	ESS	PRF	Better results in adhesion (80% none vs 56%), infection, and granulation (p<0.05).	Growth factors released by activated platelets facilitate revascularization and tissue remodeling.
Steiner et al. (2025)^4^	Double-blind RCT	72	Tympanoplasty	PRP & PRG	Significantly higher closure rate (88.9% vs 66.7%) and reduced mucosal oedema.	Reduction in oedema suggests downregulation of pro-inflammatory cytokines (Immunomodulation); extracellular vesicles promote collagen production.
Wiriyaamornchai et al. (2025)^5^	Double-blind RCT	30	Tonsillectomy	PRP	Significantly lower postoperative pain scores (Days 1-5) and faster re-epithelialization (Days 7 & 14).	Analgesic effect indicates suppression of nociceptive mediators; contains PDGF, TGF-β, IGF promoting cell proliferation.
Saini et al. (2024)^9^	Prospective Observational	80	Myringoplasty	PRF	Significantly improved graft survival rates (97.5% vs 87.5% at 20 weeks, p<0.05).	Fibrin scaffold provides mechanical support and enhances vascularization through VEGF and angiogenesis.
Mackay et al. (2024)^10^	Non-RCT Prospective	48	Vocal Fold Injection	PRP	Significant improvement in VHI-10 scores (p<0.001) and tissue function.	Regulation of tissue remodeling pathways to prevent excessive fibrosis and restore vocal fold architecture.
Reksodiputro et al. (2021)^11^	RCT	20	Total Laryngectomy	PRF	Significantly reduced postoperative oedema (p=0.002) and pain.	TGF-β triggers chemotaxis and modulates inflammation to reduce oedema; TSP-1 expressed in fibrin membrane.
Oflaz et al. (2024)^12^	Prospective, double-blind	41	Tonsillectomy	Liquid PRF	No significant effect on pain/bleeding; slightly higher healing on PRF side (34.1% vs 29.3%).	TGF-β1 theoretically accelerates soft tissue healing; modulation of local cytokine network (IL-1β, TNF-α).
Katz et al. (2025)^13^	RCT (Pilot Study)	20	Primary Palatoplasty	A-PRF+	Lower fistula rate trend (20% vs 40%), though statistical significance limited by pilot size.	Sustained release of TGF-β to recruit fibroblasts; A-PRF membranes release growth factors for 7–28 days.
Mandour et al. (2023)^15^	Prospective RCT	320	Fat Graft Myringoplasty	PRP	Significantly enhanced closure success (87,6% vs 72,7%, p=0.001) and faster healing.	Delivery of a supraphysiological pool of growth factors (PDGF, IGF, VEGF) tissue regeneration cascades. activating
Salaheldin (2012)^16^	Prospective RCT	60	Turbinate Diathermy	PRP	Improved nasal mucociliary clearance (p=0.004) and decreased crusting (6.6% vs 30%).	Synergistic action of PDGF, VEGF, and TGF-β to enhance mucociliary function and reduce inflammation

APC = autologous platelet concentrates; IGF = insulin-like growth factor; IL = interleukin; PDGF = platelet-derived growth factor; PRF = platelet-rich fibrin; PRP, platelet-rich plasma; RCT = randomised controlled trial; TGF-β = transforming growth factor-beta; TNF-α = tumour necrosis factor-α; VEGF = vascular endothelial growth factor.

APCs were applied across diverse ORL procedures, including otology (myringoplasty, tympanoplasty), rhinology (endoscopic sinus surgery) and laryngology (tonsillectomy, total laryngectomy). The introduction of recent high-quality RCTs, such as those by Steiner *et al.* (2025) and Wiriyaamornchai *et al.* (2025).^4^^,^^5^ strengthens the evidence base, particularly for outcomes related to post-operative morbidity (pain and oedema).

Clinically, APCs consistently improved key healing outcomes such as graft uptake and accelerated epithelialisation. However, the most notable and consistent clinical finding across the studies is the significant reduction in post-operative morbidity, including lower pain scores and reduced mucosal oedema.^3^^,^^9^^,^^10^ While the proliferative effect of TGF-β1 remains the known regenerative foundation, the potent clinical effect on reducing pain and oedema strongly suggests a predominant role of immunomodulation in suppressing the inflammatory microenvironment.^10^^,^^11^ Importantly, no significant adverse effects were reported across the included cohort, underscoring the safety of these autologous preparations.^1^^,^^2^^,^^9^

### Synthesis of results by otorhinolaryngology sub-specialty

#### Otology (myringoplasty and tympanoplasty)

The application of APCs in otological procedures, predominantly myringoplasty, demonstrated the most consistent evidence for enhancing healing. Of the included studies, six focused on tympanic membrane repair.^1^^,^^2^^,^^4^^,^^9^^,^^15^ The primary clinical outcome graft uptake was significantly improved with the use of APCs, particularly PRF, with success rates typically exceeding 90 per cent.^9^^,^^15^

More importantly, the high-quality RCT by Steiner *et al.* (2025) which used PRP/PRG, reported a significantly higher closure rate (88.9 per cent vs 66.7 per cent in controls) and critically, a marked reduction in mucosal oedema in the APC group during the early post-operative phase.^4^ This reduction in local inflammation strongly suggests that the benefit of APCs in the middle ear environment extends beyond mechanical regeneration, proposing an immunomodulatory role in suppressing acute inflammatory responses. No study reported differences in pure tone average (PTA) or air–bone gap (ABG) improvement that reached clinical significance, suggesting APCs primarily influence tissue healing and acute morbidity.

#### Laryngology (tonsillectomy and laryngeal procedures)

In the field of laryngology, the efficacy of APCs primarily relates to controlling post-operative morbidity and enhancing soft tissue recovery. The application in tonsillectomy, a procedure frequently associated with severe post-operative pain, demonstrated key benefits. Specifically, the high-quality randomised controlled trial by Wiriyaamornchai *et al.* (2025) using PRP, reported a significant reduction in post-operative pain scores (VAS) during the critical first five days, coupled with faster re-epithelialisation.^5^ This consistent analgesic effect supports a strong immunomodulatory role for APCs in suppressing acute inflammatory mediators at the wound bed.

For complex resective surgeries, such as total laryngectomy, PRF application enhanced the overall wound healing process, showing specific efficacy in reducing both oedema and pain in the post-operative period.^11^ Furthermore, in benign vocal pathologies, PRP injection showed promise in improving vocal fold tissue function.^10^ This benefit is hypothesised to be mediated by the anti-fibrotic properties of growth factors, which regulate excessive scar formation.^10^ Collectively, the findings in laryngology highlight the potential of APCs not just for structural tissue repair, but also for accelerating functional restoration and patient comfort.

#### Rhinology (endoscopic sinus surgery and nasal procedures)

In the field of rhinology, the application of APCs has demonstrated significant benefits in accelerating post-operative mucosal healing and reducing complications. Studies investigating endoscopic sinus surgery (ESS) found that the use of PRF can reduce adhesion formation, bleeding and granulation.^3^ This benefit is primarily attributed to the three-dimensional fibrin matrix of PRF, which acts as an ideal scaffold for sinonasal mucosal regeneration, a structure deemed suitable for the longer healing timeline of the sinus cavity.

Furthermore, in other nasal procedures such as inferior turbinate submucous diathermy, PRP application showed improvement in nasal mucociliary clearance and decreased the incidence of post-operative bleeding and crust formation.^16^ This effect underscores the advantage of PRP’s rapid growth factor release, which is more appropriate for procedures requiring quick surface epithelialisation. Overall, the findings in rhinology suggest that PRF is more beneficial in complex sinus cases (for mechanical support), while PRP is effective for faster superficial mucosal recovery.

## Discussion

This systematic review of 12 studies demonstrates that APCs are effective therapeutic adjuncts across diverse otorhinolaryngological procedures. The included studies, which encompass high-quality RCTs from 2024 and 2025, consistently highlight two major clinical benefits: the enhancement of structural tissue regeneration and, significantly, the reduction of post-operative morbidity. While previous reviews have primarily focused on graft uptake rates, our updated analysis identifies a paradigm shift towards the use of APCs as potent agents for pain and oedema control, particularly in mucosal surgeries.^4^^,^^5^

The variability in clinical outcomes across different procedures can be largely explained by the differential release kinetics of growth factors between PRP and PRF. Our analysis supports a dual-mechanism hypothesis. PRP is characterised by a rapid “burst release” of bioactive factors upon activation.^17^ This immediate release provides a high concentration of anti-inflammatory cytokines during the critical first 24–48 hours post-surgery, which correlates perfectly with the significant reduction in acute pain scores observed in tonsillectomy patients.^5^^,^^12^ In contrast, PRF contains a dense fibrin matrix that traps growth factors and allows for a slower, sustained release over 7–14 days.^18^ This prolonged release profile acts as a “biological scaffold,” which is mechanistically essential for the vascularisation and integration of tympanic grafts in myringoplasty, where the healing process requires sustained support rather than immediate symptom relief.^1^^,^^9^

Beyond growth factor release, the rapid suppression of mucosal oedema reported in recent trials strongly suggests a dominant immunomodulatory role for APCs. The observed reduction in oedema and post-operative pain challenges the traditional view that APCs function solely through proliferation. Instead, the evidence suggests that APCs actively reprogram the local inflammatory microenvironment.^11^ This is hypothesised to involve the localised downregulation of pro-inflammatory cytokines, such as IL-6, which are typically elevated in acute surgical trauma.^4^ Furthermore, the accelerated epithelialisation observed in mucosal defects suggests a balanced regulation of MMP-9, preventing excessive tissue degradation and facilitating a smoother transition from the inflammatory to the proliferative phase.^10^ Preclinical models corroborate this, showing that APC application significantly reduces inflammatory cell infiltration and oxidative stress in nasal mucosa.^6^

The aggregate data from six studies confirms that APCs, particularly PRF, significantly improve graft uptake rates (up to 97.5 per cent) compared to conventional methods.^9^^,^^15^ The fibrin matrix appears to stabilise the graft in the avascular environment of the tympanic membrane, consistent with recent meta-analytic findings.^8^ In Rhinology, the use of APCs in endoscopic sinus surgery has been shown to reduce adhesion formation and bleeding, attributed to the enhancement of mucosal re-epithelialisation.^3^ Meanwhile, in Laryngology, specifically tonsillectomy, the primary benefit is analgesic, with PRP significantly lowering pain scores and accelerating the return to normal diet.^5^

Despite these promising findings, a critical limitation identified in the current literature is the scarcity of direct molecular analysis alongside clinical outcomes. As highlighted by our systematic search analysis of 40 relevant records, there remains a substantial evidence gap regarding biomarker quantification in otorhinolaryngological APC studies. Specifically, systematic data indicate that only 12.5 per cent of studies measured IL-6, merely 5 per cent quantified TGF-β and none (0 per cent) directly measured MMP-9 levels in patient tissues. Consequently, the biological mechanisms discussed herein are theoretically inferred from clinical phenotypes rather than confirmed by direct quantification in the included human subjects. Furthermore, the lack of standardisation in preparation protocols (e.g., centrifugation speed and time) remains a barrier to reproducibility, as variations in G-force can significantly alter the leukocyte and growth factor content of the final product.^7^

What is already known on this topic

- Autologous platelet concentrates (APCs), including platelet-rich plasma (PRP) and platelet-rich fibrin (PRF), are increasingly used in otological, rhinological, and laryngological surgery to enhance graft uptake and mucosal healing
- Existing clinical studies mainly focus on structural outcomes (e.g., graft closure, epithelialization), with heterogeneous preparation protocols and limited systematic evaluation of post-operative pain, oedema or mechanistic biomarkers

What this paper adds

- This systematic review of 12 prospective and randomised studies demonstrates that autologous platelet concentrates (APCs) consistently improve tissue regeneration and, importantly, reduce post-operative morbidity, particularly pain and mucosal oedema after myringoplasty, tympanoplasty and tonsillectomy
- The synthesis supports a procedure-specific role of PRF for sustained graft support and PRP for rapid symptom control, and proposes a predominant immunomodulatory mechanism involving downregulation of interleukin-6 (IL-6) and regulation of matrix metalloproteinase-9 (MMP-9) activity
- The review identifies critical gaps in standardized APC preparation and biomarker quantification, defining clear priorities for future translational trials in otorhinolaryngology

## Conclusion

This systematic review establishes that APCs are effective adjunctive therapies in otorhinolaryngology, offering significant benefits not only for structural tissue regeneration but also for reducing post-operative morbidity. The evidence supports a procedure-specific application strategy: PRF is optimal for otological and complex sinus procedures requiring sustained scaffolding and prolonged growth factor release to ensure graft integration. Conversely, PRP demonstrates superior utility in mucosal surgeries, such as tonsillectomy and turbinate reduction, where its rapid “burst release” of bioactive factors provides immediate analgesic and anti-inflammatory effects.

Furthermore, the consistent reduction in acute mucosal oedema and pain observed in recent high-quality trials suggests that APCs function through a potent immunomodulatory mechanism, likely involving the downregulation of pro-inflammatory cytokines like IL-6 and the regulation of MMP-9 activity. While the safety profile of APCs is excellent, future clinical practice and research must prioritise the standardisation of preparation protocols and the systematic quantification of biomarkers to maximise therapeutic reproducibility and validate these molecular pathways.

## PROSPERO registration statement

This systematic review has been registered in the International Prospective Register of Systematic Reviews (PROSPERO) under the registration number CRD420251137161.
